# Serum Levels of MDC and MMP-9 and the Relationship Between Serum Levels and Disease Activity in the Patients with Systemic Lupus Erythematosus

**DOI:** 10.12669/pjms.314.7325

**Published:** 2015

**Authors:** Yang Liu, Ning Tie, Lijie Bai

**Affiliations:** 1 Yang Liu, PhD Department of Rheumatology, the First Affiliated Hospital, Inner Mongolia Medical University, China; 2 Ning Tie, M.S Department of Rheumatology, the First Affiliated Hospital, Inner Mongolia Medical University, China; 3 Li-Jie Bai, M.S Department of Rheumatology, the First Affiliated Hospital, Inner Mongolia Medical University, China

**Keywords:** Lupus erythematosus, Systemic, Macrophage-derived chemokine, Matrix metalloproteinase-9

## Abstract

**Background and Objective:**

Systemic lupus erythematosus (SLE) is a complicated autoimmune disease. Although its pathogenesis is not clear, cytokine may be involved in it. So we investigated serum levels of macrophage-derived chemokine (MDC), and matrix metalloproteinase-9 (MMP-9), and to determine the relationship between serum levels and the disease activity of SLE.

**Methods:**

Serum levels of MDC and MMP-9 were measured by enzyme-linked immuno sorbent assay (ELISA).

**Results:**

Significantly decreased serum levels of MDC and MMP-9 were found in SLE as compared to those in controls (P<0.001 P<0.001), but serum levels of MDC and MMP-9 increased after treatment (P<0.001 P<0.05). Serum levels of MDC and MMP-9 were lower in patients with active disease than those with inactive disease (P<0.001 P<0.05). Significantly decreased serum levels of MDC and MMP-9 were found in patients with renal damage than those without the damage (P<0.001 P<0.05). Serum level of MDC was lower in patients with arthritis than those without the damage (P<0.001), but serum level of MMP-9 has no significant difference in two groups (P>0.05).

**Conclusion:**

The present data suggest that MDC and MMP-9 may be involved in the pathogenesis of SLE, and serum levels of MDC and MMP-9 could be markers of monitoring disease activity, renal damage, disease progression and improvement in SLE.

## INTRODUCTION

Systemic lupus erythematosus (SLE) is an autoimmune disease characterized by the increased production of autoantibodies by systemic clinical manifestation and damage to multiple organs. This study aimed at analysing the relationship between serum levels of macrophage-derived chemokine (MDC), and matrix metalloproteinase-9 (MMP-9) and disease activity of patients with SLE in order to evaluate their roles in the pathogenesis and course of the disease as well as their diagnostic values.

## METHODS

### Subjects

Diagnosis of SLE was established according to the 1997 revised American Rheumatism Association Criteria^1^, and disease activity was evaluated by the SLEDAI (SLE Disease Activity Index).^2^ The patients with SLE (32 women and 4 men, aged 11-61 yrs) were divided into 2 groups: 13 SLE patients with renal disease and 23 SLE patients without renal disease according to proteinuria (++↑ or >0.5g/d); 23 active course patients and 13 inactive course patients were evaluated using the SLEDAI standard, and active disease was indicated by SLEDAI score of more than 8 points; 23 SLE patients with arthritis and 13 without the lesion according to articular damage. 30 sex and age matched healthy volunteers were recruited as controls.

### Serum

Three milliliters of venous peripheral blood were collected from each subject, centrifuged to get serum, and then stored at -80ºC. Nine serum samples were collected from patients taking corticosteroid three or four weeks later. The concentrations of MDC and MMP-9 were measured by ELISA respectively. ELISA kits for HGF, MDC and MMP-9 were purchased from R&D Systems Inc (Catalog No: DMD00, DMP00). Assays were performed according to the manufacturer’s instructions.

### Statistical analysis

All the results were presented as mean±standard deviatioin. The differences were tested for statistical significance by Student’s *t* test and relative risk. A probability (*P*) less than 0.05 was considered as significantly different.

## RESULTS

Significantly decreased serum levels of MDC and MMP-9 was found in SLE patients as compared to those in controls, 450.95±76.76 pg/ml vs 606.23±23.71 pg/ml (*P*<0.001) and 108.52±113.23 ng/ml vs 352.25±155.01ng/ml (*P*<0.001) respectively. Serum levels of MDN and MMP-9 were markedly lower in active patients than those with inactive disease, namely 393.98±53.73pg/ml vs 555.40±196.07 pg/ml (*P*<0.001), and 71.70±66.24 ng/ml vs165.80±145.82 ng/ml (*P*<0.05) respectively. Serum levels of MDC and MMP-9 were decreased in patients with renal damage than those without the damage, 366.81±56.25 pg/ml vs 496.85±114.55 pg/ml (*P*<0.001), and 72.08±56.31ng/ml vs 141.93±140.55 ng/ml (*P*<0.05) respectively. Markedly lower serum level of MDC was found in patients with arthritis than those without the damage, 386.43±48.79 pg/ml vs 569.25±199.47 pg/ml (*P*<0.001), but serum level of MMP-9 has no significant difference in these two groups (*P*>0.05). Relative analysis showed that no positive correlation was found among the serum level of MDC and SLEDAI (*r* =0.205 *P*>0.05), but serum level of MMP-9 showing a negative correlation with SLEDAI (*r* = −0.41 *P*<0.01), as Table-I and Table-II.

**Table-I:** The comparison of serum level of MDC between SLE patients and controls

Group	n	x̄±s (pg/ml)	t value	P value
Controls	30	606.23±23.71	10.63	<0.001
SLE patients	36	450.95±76.76		
Renal damage	13	366.81±56.25	3.68	<0.001
No renal damage	23	496.85±114.55		
Active	23	393.98±53.73	3.72	<0.001
Inactive	13	555.40±196.07		
SLEDAI>8	23	389.46±57.88	3.69	<0.001
SLEDAI≤8	13	538.81±168.29		
Arthritis	23	386.43±48.79	4.13	<0.001
No arthritis	13	569.25±199.47		
Before treatment	9	163.91±48.36	6.72	<0.001
After treatment	9	452.78±102.99		

**Table-II:** The comparison of serum level of MMP-9 between SLE patients and controls.

Group	n	x̄±s (ng/ml)	t value	P value
Controls	30	352.25±155.01	7.92	<0.001
SLE patients	36	108.52±113.23		
Renal damage	13	72.08±56.31	2.17	<0.05
No renal damage	23	141.93±140.55		
Active	23	71.70±66.24	2.98	<0.05
Inactive	13	165.80±145.82		
SLEDAI>8	23	80.29±71.61	2.20	<0.05
SLEDAI≤8	13	152.45±149.80		
Arthritis	23	103.37±126.35	0.40	>0.05
No arthritis	13	117.32±89.40		
Before treatment	9	114.41±92.40	2.00	<0.05
After treatment	9	245.64±196.57		

## DISCUSSION

SLE is a complicated autoimmune disease characterized by various immunological abnormalities, including polyclonal activation of circulating B lymphocyte that produce a large quantity of autoreactive antibodies, the abnormality of T lymphocyte and IC deposition. During the process, many chemokines and their receptors play an important regulating role.^3^

If monocyte/macrophage releases IL-10 continuously, it will result in division of Th cell to Th2 cell. If monocyte/macrophage releases IL-12 continuously, it will promote transformation of Th cell to Th1 cell. So we focus on cytokines derived from monocyte/macrophage and this will demonstrate the role of chemokines in SLE pathogenesis.

MDC, a CC chemokine, is a potent chemoattractant which activated Th2 lymphocytes via the chemokine receptor CCR4,^4^ and its receptor CC chemokine receptor 4 (CCR4) preferentially expressed on Th2 cells. Th2 type cytokines IL-4 and IL-13 downregulated the production of MDC. This may partially contribute to maintaining Th1/Th2 balance.^5^ Dendritic cells, B lymphocytes and macrophages all produced MDC constitutively, while NK cells, monocytes, and CD4^+^ T lymphocytes produce MDC upon stimulation. IFN-γ can also suppress MDC expression in monocyte, macrophage, and dendritic cells.^6^ Because many immune cells can produce MDC and MDC can chemoattract many immune cells, MDC may be essential in SLE pathogenesis. Serum level of MDC in 36 SLE patients was tested and it was discovered that serum level of MDC was significantly decreased in patients as compared to those in controls, and markedly decreased in patients with active disease than those without these diseases. All these demonstrated MDC was involved in SLE process, it might be relevant to SLE activity, and it might be a new sensitive marker. Dolff^7^ reported that SLE was a Th2 type disease, but our results showed the significantly decreased serum level of MDC. Whether SLE is not the dominant Th2 type disease remains to be further studied.

MMP-9 was involved in inflammation and immune system dysfunctions. Besides immunologic abnormalities, SLE also presents chronic inflammatory components. Therefore, a role of MMP-9 in SLE pathology might be suspected.^8^ Serum level of MMP-9 in 36 SLE patients was tested and it was discovered that serum level of MMP-9 was significantly decreased in patients as compared to those in controls, and was lower in patients with active disease than those with inactive disease. A negative relation between serum level of MMP-9 and SLEDAI was found, which may reflect dynamic change of SLE.

SLE tends to damage multiple organs, including the kidney and article. Our test showed the serum levels of MDC and MMP-9 were decreased in patients with renal damage than those without the damage, and the serum level of MDC was markedly decreased in patients with arthritis than those without the damage. So MDC and MMP-9 may be involved in the pathogenesis of lupus nephritis and seemed to be markers of renal damage, and MDC may be involved in the pathogenesis of arthritis and seemed to be a new sensitive marker of article damage. As Garcia^9^ and his colleagues reported, they found that MDC was critically involved in the development at anti-GBM GN from acute glomerular injury to irreversible tissue damage. Sato^10^ and his colleagues investigated the serial changes of glomerular metalloproteinase activity in antithymocyte-induced glomerulonephritis in rats and found attennated glomerular MMP-9 activity.

The serum levels of MDC and MMP-9 in 9 SLE patients taking corticosteroid 3 or 4 weeks later were examined and it was found that clinical symptoms were relieved, indexes of lab test improved, serum level of MDC and MMP-9 increased. The increased serum level of MDC remains to be studied, but the increased serum level of MMP-9 may explain that glucocortisone influenced the release of MMP-9 from blood circulation.^11^ It is thought that the increased serum level of MDC and MMP-9 may demonstrate the lupus improvement, so the serum levels of MDC and MMP-9 may serve as markers for the determination of disease progression or improvement.

MDC and MMP-9 were involved in the pathogenesis of SLE. What is their relationship remains to be studied. Vermaelen^12^ and his colleagues reported that the specific absence of MMP-9 activity inhibited the development of allergic airway inflammation by impairing the recruitment of DCs into airways and the local production of DC-derived chemokines. At present, DCs are the most powerful antigen presenting cells and the main resource of MDC.^13^ The functional disorder antigen presenting cells^14^ and the reduced DCs^15^ are in existence in patients with SLE has been reported and the proportion of DCs in peripheral blood showed a negative correlation with disease activity.^15^ From our study it has been shown that serum levels of MDC and MMP-9 were significantly decreased in SLE patients. Whether this mechanism may also be involved in SLE pathogenesis, it depends on further accumulation of clinical researches.

## CONCLUSION

SLE is an autoimmune disease. The useful measurement to cure this disease is Prednisone. When symptom ease, the dosage of Prednisone should be reduced. At this time, patient’s condition prefers to relapse, especially during 10-15mg a day to take. So the combing between Traditional and Western medicine is very important. The combination can control its deterioration and reduce its relapse so it is better to cure it.

### Limitations of the Study

Small sample size was one of the important limitations of this study.

## References

[1] (1999). Arthritis & Rheumatism. Guidelines for referral and management of systemic lupus erythematosus in adults. American College of Rheumatology Ad Hoc Committee on Systemic Lupus Erythematosus Guidelines.

[2] Bombardier C, Gladman DD, Urowitz MB, Caron D, Chang CH, Austin A (1992). Derivation of the SLEDAI disease activity index for lupus patients. The Committee on Prognosis Studies in SLE. Arthritis Rheum.

[3] Moser K, Kalies K, Szyska M, Humrich JY, Amann K, Manz RA (2012). CXCR3 promotes the production of IgG1 autoantibodies but is not essential for the development of lupus nephritis in NZB/NZW mice. Arthritis Rheum.

[4] Ishizaki S, Kasuya Y, Kuroda F, Tanaka K, Tsuyusaki J, Yamauchi K (2012). Role of CD69 in acute lung injury. Life Sci.

[5] Kwon DJ, Bae YS, Ju SM, Goh AR, Youn GS, Choi SY (2012). Casuarinin suppresses TARC/CCL17 and MDC/CCL22 production via blockade of NF-κB and STAT1 activation in HaCaT cells. Biochem Biophys Res Commun.

[6] Yoon WS, Ryu SR, Lee SS, Chae YS, Kim EJ, Choi JH (2012). Suppression of inflammation by recombinant Salmonella typhimurium harboring CCL22 microRNA. DNA Cell Biol.

[7] Dolff S, Bijl M, Huitema MG, Limburg PC, Kallenberg CG, Abdulahad WH (2011). Disturbed Th1, Th2, Th17 and T(reg) balance in patients with systemic lupus erythematosus. Clin Immunol.

[8] Wu Q, Yang Q, Lourenco E, Sun H, Zhang Y (2001). Interferon-lambda1 induces peripheral blood mononuclear cell-derived chemokines secretion in patients with systemic lupus erythematosus: its correlation with disease activity. Arthritis Res Ther.

[9] Garcia GE, Xia Y, Harrison J, Wilson CB, Johnson RJ, Bacon KB (2003). Mononuclear cell-infiltrate inhibition by blocking macrophage-derived chemokine results in attenuation of developing crescentic glomerulonephritis. Am J Pathol.

[10] Sato Y, Fujimoto S, Hamai K, Eto T (1998). Serial alterations of glomerular matrix-degrading metalloproteinase activity in anti-thymocyte-induced glomerulonephritis in rats. Nephron.

[11] Gheorghe KR, Sadique S, Leclerc P, Idborg H, Wobst I, Catrina AI (2012). Limited effect of anti-rheumatic treatment on 15-prostaglandin dehydrogenase in rheumatoid arthritis synovial tissue. Arthritis Res Ther.

[12] Vermaelen KY, Cataldo D, Tournoy K, Maes T, Dhulst A, Louis R (2003). Matrix metalloproteinase-9-mediated dendritic cell recruitment into the airway is a step in a mouse model of asthma. J Immunol.

[13] Kuroda E, Sugiura T, Okada K, Zeki K, Yamashita U (2001). Prostaglandin E2 up-regulates macrophage-derived chemokine production but suppresses IFN-inducible protein-10 production by APC. J Immunol.

[14] Tucci M, Stucci S, Strippoli S, Silvestris F (2010). Cytokine overproduction, T-cell activation, and defective T-regulatory functions promote nephritis in systemic lupus erythematosus. J Biomed Biotechnol.

[15] Duffau P, Blanco P (2012). Can dendritic cells still be tamed in systemic lupus erythematosus? Clin Immunol.

